# High-throughput sequencing reveals small RNAs involved in ASGV infection

**DOI:** 10.1186/1471-2164-15-568

**Published:** 2014-07-07

**Authors:** Marike Visser, Hans J Maree, D Jasper G Rees, Johan T Burger

**Affiliations:** Biotechnology Platform, Agricultural Research Council, Pretoria, South Africa; Genetics Department, Stellenbosch University, Stellenbosch, South Africa; Infruitec-Nietvoorbij, Agricultural Research Council, Stellenbosch, South Africa

**Keywords:** *Apple stem grooving virus*, Next-generation sequencing, Plant-virus interaction, tRNA-derived fragment, tRNA-half, Virus-derived small interfering RNA

## Abstract

**Background:**

Plant small RNAs (sRNAs) associated with virulent virus infections have been reported by previous studies, while the involvement of sRNAs in latent virus infection remains largely uncharacterised. Apple trees show a high degree of resistance and tolerance to viral infections. We analysed two sRNA deep sequencing datasets, prepared from different RNA size fractions, to identify sRNAs involved in *Apple stem grooving virus* (ASGV) infection.

**Results:**

sRNA analysis revealed virus-derived siRNAs (vsiRNAs) originating from two ASGV genetic variants. A vsiRNA profile for one of the ASGV variants was also generated showing an increase in siRNA production towards the 3′ end of the virus genome. Virus-derived sRNAs longer than those previously analysed were also observed in the sequencing data. Additionally, tRNA-derived sRNAs were identified and characterised. These sRNAs covered a broad size-range and originated from both ends of the mature tRNAs as well as from their central regions. Several tRNA-derived sRNAs showed differential regulation due to ASGV infection. No changes in microRNA, natural-antisense transcript siRNA, phased-siRNA and repeat-associated siRNA levels were observed.

**Conclusions:**

This study is the first report on the apple sRNA-response to virus infection. The results revealed the vsiRNAs profile of an ASGV variant, as well as the alteration of the tRNA-derived sRNA profile in response to latent virus infection. It also highlights the importance of library preparation in the interpretation of high-throughput sequencing data.

**Electronic supplementary material:**

The online version of this article (doi:10.1186/1471-2164-15-568) contains supplementary material, which is available to authorized users.

## Background

The domesticated apple, *Malus* x *domestica* (*M*. x *domestica*), has a wide range of infectious agents, which include fungi, bacteria, phytoplasma, viruses and viroids. One such virus, *Apple stem grooving virus* (ASGV), is the type member of the genus *Capillovirus* (family *Flexiviridae*) [1]. It is a positive-sense RNA virus with a genome of approximately 6.5 kb, which is organised into two overlapping open reading frames (ORFs) [2]. ASGV infection is mostly symptomless (latent) in apple cultivars, depending on the virus strain, however some cultivars are susceptible and may develop severe symptoms such as xylem pitting and grooving, phloem necrosis and the complete decay of the tree [3].

During infection the replication of RNA viruses generate long dsRNA intermediate molecules that triggers the synthesis of small interfering RNAs (siRNAs) [4]. Furthermore, the folded duplex regions of single stranded viral RNAs can also result in siRNA synthesis [5]. These virus-derived siRNAs (vsiRNAs) subsequently regulate viral RNA expression through a process known as RNA silencing. In addition to vsiRNA production, plants’ endogenous small RNA (sRNA) pathways are also affected by viral infection [6–8].

With the introduction of next-generation sequencing the knowledge of sRNA species has been extended beyond the well-characterised miRNA, trans-acting siRNA (tasiRNA) and natural-antisense transcript (NAT) siRNA (nat-siRNA) groups. Although sRNAs were shown to originate from tRNA before, Lee et al. [9] was the first to illustrate that these molecules were not produced by non-systematic tRNA degradation [9]. Small RNAs associated with tRNAs have been divided into two categories based on the tRNA region they originate from. The first group, called tRNA halves (tsRNA/tiRNA), are derivatives of mature tRNAs cleaved in the anticodon loop, resulting in functional sRNAs of around 28 to 36 nucleotides in size. Enzymes involved in their biogenesis have been identified for humans [10], yeast [11] and bacteria [12], but are still unknown in plants.

Transfer RNA cleaved in the D or T loop give rise to a second group of sRNAs, called tRNA-derived RNA fragments (tRFs). This group can be further divided into sRNAs stemming from (a) the 5′ end of mature tRNAs, (b) the 3′ end of mature tRNAs and (c) the 3′ end of immature tRNAs, called 5′-tRFs, 3′ CCA tRFs and 3′ U tRFs respectively [13]. Several synonyms have been used for the different sub-groups [9, 14].

In this study a next-generation sequencing approach was followed to identify sRNAs that are associated with a latent virus infection in apple plants. In addition to illustrating the vsiRNA profiles associated with an ASGV genetic variant the results from this study demonstrate the involvement of tRNA-derived sRNAs in plant-virus interaction. The lack of differential regulation of miRNAs, phasiRNAs, nat-siRNAs and rasiRNAs in leaf material is also shown.

## Results and discussion

### sRNA sequencing libraries

**Figure 1:**
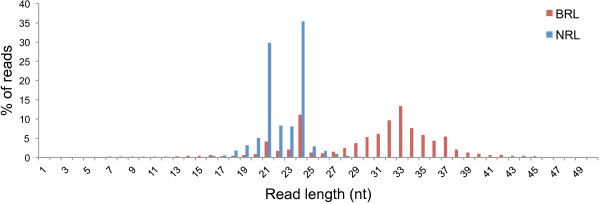
**Size-distribution of the two sRNA sequencing library types.** Histogram illustrating the number of reads, 1 to 50 nt in length, as a percentage of the reads in this size-range for the BRL and NRL data respectively.

Two library preparation approaches were followed. The first approach made use of total RNA to produce a broad range library (BRL) for each sample, with individual sequencing datasets comprising of between 7,543,861 and 11,648,479 reads. Reads of 27 nt and longer contributed to 73% of all BRL reads. Since sRNAs involved in gene silencing are mostly considered to fall within the 17 to 26 nt size-range, a second narrow range library (NRL) was prepared for each sample using size-selected sRNAs to increase the sequence depth of these sRNAs. These libraries generated 7,235,867 to 14,896,610 high quality reads per sample. The size-range 17 to 26 nt in length represented 97% of all the reads and were used for downstream analysis. Figure 1 illustrates the size distribution of the sRNA reads (1 to 50 nt in size) for the pooled BRL and pooled NRL datasets. The histogram not only highlights the increase in the percentage of reads 17 to 26 nt in length in the NRL datasets when compared to the BRL datasets, but also shows a change in ratios between the different size groups, in particular when comparing the ratio between the 21 and 24 nt groups. Since the same total RNA extract was used to prepare both libraries, this observation demonstrates the effect of library preparation on the final sequencing data and highlights the difficulty of comparing data generated by different protocols.

### vsiRNAs resulting from ASGV infection

**Figure 2:**
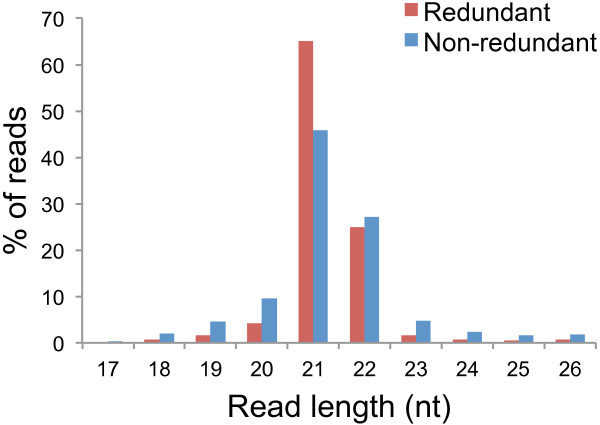
**Size-distribution of NRL vsiRNA reads.** Histogram illustrating the number of NRL vsiRNA reads, 17 nt to 26 nt in length, from the virus-infected samples, as a percentage of the reads in this size-range.

The NRL data was first used to analyse the production of vsiRNAs, since these datasets were enriched for sRNAs in the size range known to be associated with vsiRNAs. Reads, which did not align to the apple nuclear, chloroplast or mitochondrial genomes, were mapped (allowing a single mismatch) onto the complete genomes of six ASGV isolates. sRNA read-mapping results for the pooled NRL sequencing data from the virus-infected samples are shown in Table 1. In total, 0.59% of all non apple-derived reads (17 to 26 nt in length) from the infected samples mapped onto at least one of the ASGV genomes. The large number of reads mapped onto the genome of the German isolate ASGV-AC, with 98% coverage. The majority of the virus-derived reads from the NRL data were 21 nt long followed by reads 22 nt in length (Figure 2), which is often seen for positive-sense RNA viruses [15, 16].

**Table 1:** **Results for the virus-infected NRL sRNA read-mapping against ASGV genomes**

Isolate	GenBank Accession number	Country	Host	Genome size (nt)	Total number of reads mapped	Non-redundant number of reads mapped	Genome coverage (%)
ASGV-AC	JX080201.1	Germany	*M. domestica*	6496	27069	5897	98.04
ASGVp12	HE978837.1	India	*M. domestica*	6478	25256	5297	96.34
ASGV P-209	NC_001749.2	Japan	*M. domestica*	6495	14341	3810	88.96
ASGV	D14995.2	Japan	*M. domestica*	6495	14341	3810	88.96
ASGV-HH	JN701424.1	China	*Pyrus pyrifolia*	6496	8591	1872	54.63
ASGV-CHN	JQ308181.1	China	*M. domestica*	6495	6555	1659	52.73

**Figure 3:**
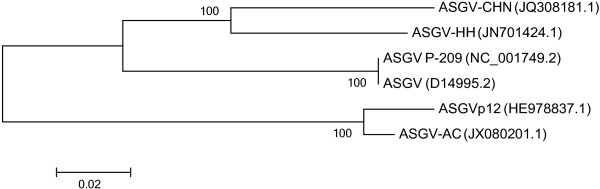
**Phylogenetic tree based on the complete genome sequence of ASGV isolates.** A neighbour joining method was applied and 1000 bootstrap replicates were used for the calculation of branch support. The branch length represents the number of substitutions per nucleotide position is indicated by the scale bar.

**Figure 4:**
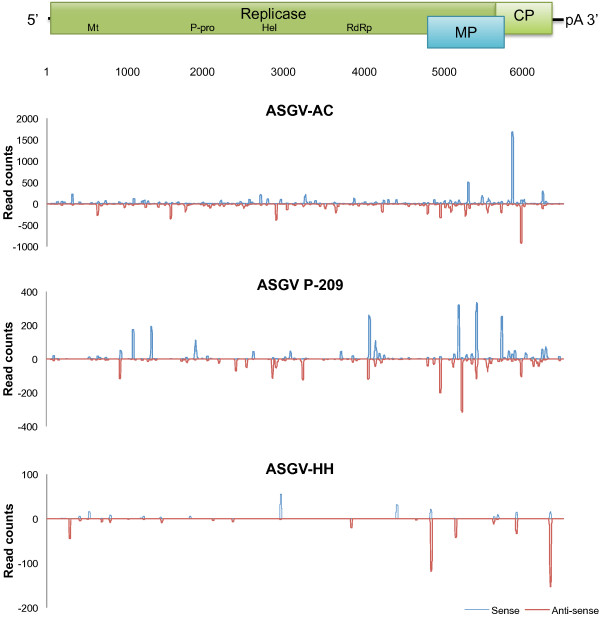
**Distribution of variant-specific sRNA reads along ASGV genomes.** The genomic positions of reads that could only map onto the genome of a single ASGV isolate are illustrated. Reads that mapped onto the positive or negative strand of the virus are represented in blue and red respectively. A schematic representation of the genome above the graphs illustrates the genomic positions of the vsiRNA reads.

The occurrence of mixed ASGV infection was analysed using the genomes of three isolates (ASGV-AC, ASGV P-209 and ASGV-HH). These isolates each had an equal or higher sRNA read count (Table 1) than their closest relative (Figure 3). To determine the sRNA reads associated only with a specific variant genome, reads with a uniquely mapped position and genome were reported (Table 2). The large majority of variant-specific reads were associated with ASGV-AC, followed by the Japanese isolate (P-209). These variant-specific reads were distributed along the length of each of the three genomes (Figure 4), indicating that more than one ASGV variant was present with distinct genome sequences, rather than a single recombinant virus. Given their reasonably large number of total, as well as variant-specific reads, we suggest that at least two ASGV genetic variants, closely related to ASGV-AC and P-209 respectively, were present in the samples. Closer assessment of the read-mapping profiles for the individual samples suggested that two samples contained a mixed infection of the two variants, while the third was singly infected with a genetic variant of ASGV-AC.

**Table 2:** **Results for the vsiRNA variant-specific read-mapping**

Isolate	GenBank Accession number	ASGV-infected sample 1	ASGV-infected sample 2	ASGV-infected sample 3	All ASGV-infected samples
ASGV-AC	JX080201.1	4074 (1326)	4905 (1544)	6507 (1832)	15486 (3111)
ASGV P-209	NC_001749.2	2469 (768)	2448 (703)	83 (20)	5000 (1120)
ASGV-HH	JN701424.1	269 (95)	267 (118)	141 (34)	677 (191)

The number of reads which mapped only onto a specific ASGV genome for each virus-infected sample as well as for the pooled data are shown. The non-redundant read counts are given within brackets.

**Figure 5:**
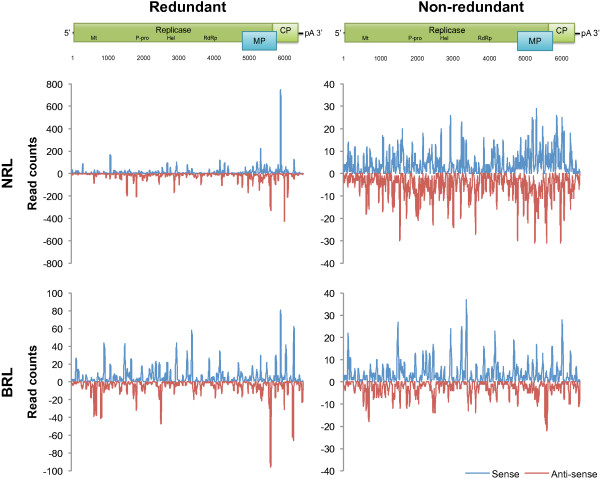
**NRL and BRL vsiRNA profiles of an ASGV-AC genetic variant.** vsiRNA profiles generated from redundant and non-redundant reads of both NRL and BRL datasets are depicted. Reads that mapped onto the positive or negative strand of the virus are represented in blue and red respectively. A schematic representation of the genome above the graphs illustrates the genomic position of the vsiRNA reads.

Since only one sample was confirmed to be infected with a single ASGV variant, only reads from this sample could be used to generate a complete vsiRNA profile of this variant. Figure 5 shows the mapping distribution of the vsiRNA reads along the virus genome. In general the 3′ end of the genomes showed regions of higher genome coverage by vsiRNAs. The increase in vsiRNAs production toward the 3′ end of the genome has previously been ascribed to the presence of viral subgenomic RNAs (sgRNAs) [17, 18]. Both the ASGV movement and coat proteins are expressed from 3′ sgRNAs [19–21] and may explain the increase in vsiRNA originating from the 3′ end. The non-redundant reads were also plotted onto the ASGV genomes (Figure 5). The majority of the genome sequences were associated with the production of more than one unique vsiRNA, illustrating that multiple Dicer-like (DCL) cleavage sites are in close proximity to each other on a virus genome.

**Figure 6:**
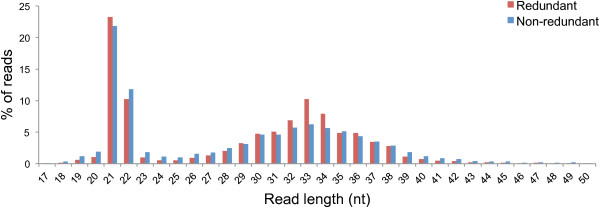
**Size-distribution of BRL vsiRNA reads.** Histogram illustrating the number of BRL vsiRNA reads, 17 nt and longer, from the virus-infected samples as a percentage of the reads in this size-range.

Altogether, from the three BRL datasets of the infected samples, 0.24% of the non apple-derived reads (>16 nt in length) mapped onto the six ASGV genomes. Although less than the NRL viral reads, these reads still covered 97% and 82% of the ASGV-AC and P-209 genomes, respectively (Table 3). Similar to the NRL datasets, the 21 nt long reads also dominated the viral reads in the BRL datasets (Figure 6). The second most abundant size group was the 22-nt group, closely followed by reads 33 nt in length. To our knowledge this is the first report of plant virus-derived sRNA reads larger than 30 nt in length. These larger sRNAs contributed significantly to the number of virus-associated reads and may point towards their biological importance. Alternatively, these reads possibly represent remnants of siRNA-directed ASGV genome degradation. The distribution of BRL reads along the ASGV genome was also examined (Figure 5). The presence of a substantial number of larger sRNAs in the BRL data resulted in a change in the vsiRNA profiles. The dominant areas of higher coverage by the conventional vsiRNAs (as can been seen from the NRL data) are surpassed (in the BRL data) by the additional areas of higher coverage, which are generated by the longer vsiRNAs. Furthermore, the 3′ vsiRNA bias was also less evident in the BRL data, compared to the NRL data. This observation once again demonstrates the effect of library preparation on sequencing results and the interpretation thereof.

**Table 3:** **Results for the virus-infected BRL sRNA read-mapping against ASGV genomes**

Isolate	GenBank Accession number	Genome size (nt)	Total number of reads mapped	Non-redundant number of reads mapped	Genome coverage (%)
ASGV-AC	JX080201.1	6496	7795	3751	97.44
ASGVp12	HE978837.1	6478	6628	3201	93.96
ASGV P-209	NC_001749.2	6495	4456	2059	82.03
ASGV	D14995.2	6495	4456	2059	82.03
ASGV-HH	JN701424.1	6496	1945	886	42.13
ASGV-CHN	JQ308181.1	6495	1341	721	39.98

### tRNA-derived sRNAs show differential regulation due to ASGV infection

**Figure 7:**
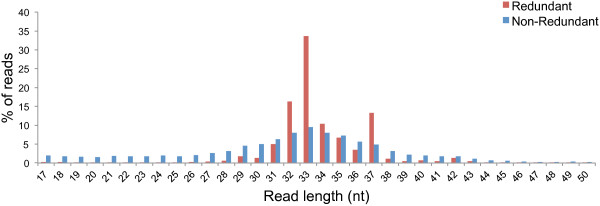
**Size-distribution of BRL tRNA-derived sRNA reads.** Histogram illustrating the number of BRL tRNA-derived sRNA reads, 17–50 nt in length, as a percentage of the reads in this size-range.

Previous studies have established that sRNAs are generated from tRNAs in a non-random manner and that they play a regulatory role similar to other sRNA species [9, 22, 23]. In the BRL data the tRNA-derived sRNAs represented 23% and 19% of the reads larger than 16 nt for the infected and healthy samples respectively. These sRNAs varied in size from 17 to 59 nt in length, representing both tRFs and tRNA-halves. The broad size-range of the tRNA-derived sRNA reads in this study demonstrates that it is not always possible to clearly distinguish between these two classes only based on sequence length and origin. The larger species also stretched beyond the recognized tRNA-half size-range, spanning the anticodon loop, similar to previous reports [24, 25]. The majority of tRNA-derived sRNAs in apple were 33 nt in length followed by reads of 32 and 37 nt (Figure 7). The dominant single tRNA-derived sRNA was a 5′ tRNA-half (33 nt long) originating from tRNA-Asp^GTC^, and was represented by a total of 1,814,310 reads in the BRL datasets. In contrast, for the NRL dataset only 1.6% of all reads (17 tot 26 nt), originated from tRNAs.

sRNAs, originating from both 5′ and 3′ ends of mature tRNAs, were identified in datasets from both library types (Additional file 1: Table S1 and S2). Additionally, and in agreement with previous studies [23, 26], sRNAs were also identified originating from the central part of tRNAs. These internal species were especially prominent in the cluster of sRNAs (in the BRL data) spawning from tRNA-Gln^CTG^.

**Figure 8:**
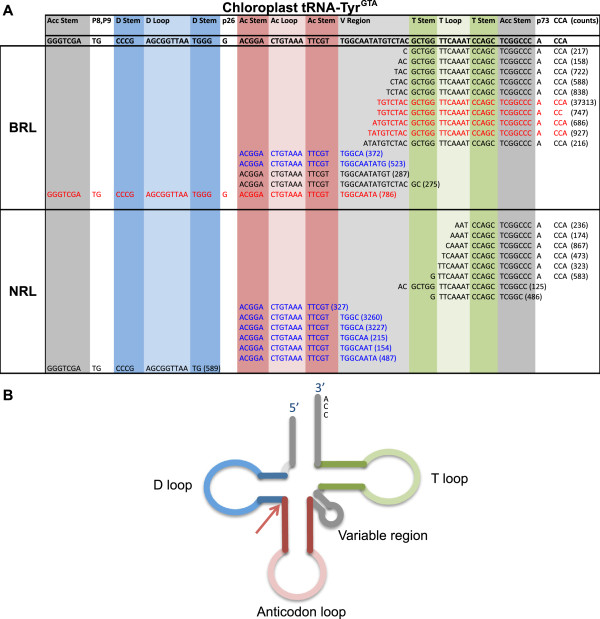
**Variation in tRNA-derived sRNA profiles. A)** Diagram showing the sRNA reads with the highest read count for each of the two types of data, which are associated with the chloroplast tRNA-Tyr^GTA^. The sRNAs, which were up- or down-regulation due to ASGV infection, are indicated in red and blue respectively. The total read count of each sRNA is indicated within brackets. **B)** The red arrow illustrates the 5′ start position of a cluster of central sRNAs, originating from tRNA-Tyr^GTA^, which are down-regulated during ASGV infection.

When the potential involvement of tRNA-derived sRNAs in ASGV infection was investigated, several tRNA-derived sRNAs showed significant variation in expression levels between infected and healthy samples (Additional file 2: Table S3 to S6). Not only did individual sRNAs show differential expression, but the total number of sRNAs spawned by some of these tRNAs was found to significantly vary between the two groups. One tRNA, tRNA-Tyr^GTA^, in particular displayed an interesting altered sRNA arrangement in the ASGV-infected samples (Figure 8). The BRL data revealed an increase in sRNAs derived from its 3′ end, extending into the variable region, while both BRL and NRL datasets showed a decrease in sRNAs that were generated from the central part of the tRNA. The 5′ ends of these internal sRNAs consistently coincided with the 5′ ends of the anticodon stems and extended into the variable regions. Due to the uniformity of their 5′ ends, these internal sRNAs cannot originate as a result of random tRNA degradation. From the inverse regulation of the two fragment types, it can be argued that a single tRNA molecule did not give rise to both species, but rather that they were generated through separate pathways. However, the possibility exists that these two processes are linked and that the production of the one species affects that of the other.

The biogenesis of tRNA-derived sRNAs, as well as the way in which they affect other molecular pathways remains to be elucidated. Earlier reports speculated that tRFs bind, to ribosomes resulting in a down-regulation of gene expression [27]. Through their association with argonaute proteins a possible role in post-transcriptional gene silencing was also suggested [23]. The biological function of the differentially regulated tRNA-derived sRNAs in the current study remains to be determined.

### The involvement of other endogenous sRNAs in ASGV infection

Besides the vsiRNAs and tRNA-derived sRNAs involved in ASGV infection, differential expression analysis showed no variation in phasiRNA and miRNA levels as a result of ASGV infection; neither did the nat-siRNAs or rasiRNAs show any change in expression levels (Additional file 2: Table S7 to S17). In addition to their regulatory role during plant development, miRNAs are often linked to stress response. The latent nature of ASGV may therefore explain what seems to be a lack of miRNA involvement during ASGV infection.

## Conclusions

In this study next-generation sequencing of sRNAs was used to investigate plant responses to latent virus infection. Two different sRNA libraries were generated per sample. Both datasets illustrated the synthesis of virus-derived sRNAs in response to ASGV infection. Along with earlier reported tRNA-derived sRNAs of more than 30 nt in length, BRL data from this study additionally suggested virus-derived RNAs larger than the well-characterised vsiRNAs of around 21 nt. The vsiRNA profiles varied depending on the method of library preparation used, illustrating the importance of consistency when comparing different samples. Additionally, the results showed that ASGV-infection resulted in a change in the expression of tRNA-derived sRNAs, although the biological function of these sRNAs remains to be elucidated. This study is the first to report on sRNAs involved in ASGV-infection in the domesticated apple.

## Methods

### Sequencing library construction and data preparation

Sample material was collected from three healthy and three asymptomatic ASGV-infected (as confirmed by RT-PCR), greenhouse-grown, *M*. x *domestica* cv. ‘Golden Delicious’ (NIVV) seedlings, grafted onto MM.109 rootstocks. The viral status was confirmed by two multiplex RT-PCR reactions described in Menzel et al. [28]. The primers for *Apple mosaic virus* detection were replaced with those described in Hassan et al. [29]. See Additional file 3: Table S18 for primer information. Total RNA was extracted from mature leaf material using the Plant RNA Reagent Kit (Invitrogen) and used for library (BRL) preparation by means of the TruSeq Small RNA library preparation kit from Illumina. For each sample a second library (NRL) was prepared using the small RNA fraction (17–29 nt) purified from total RNA using a 15% TBE-urea polyacrylamide gel. The final BRL and NRL libraries were size-selected by means of a 3% Pippin Prep cassette (Sage) and a 6% polyacrylamide gel (Invitrogen), respectively, and sequenced on an Illumina HiScan SQ instrument. The software cutadapt (V 1.0) [30] was used to remove adapter sequences and the reads were filtered for quality (phred score ≥ 20) using FASTX-toolkit (V 0.0.13) [31]. For the NRL, reads less than 17 or longer than 26 nt in length were discarded, while all filtered reads 17 nt and longer were used for the analysis of the BRL data.

### vsiRNA analysis

Reads from the three NRL virus-infected datasets were combined for vsiRNA analyses. Reads that could map with less than two mismatches onto the apple nuclear, chloroplast or mitochondrial genomes, obtained from the Genome Database for Rosaceae [32, 33], were removed. Bowtie (V 0.12.7) [34] was used to perform all read-mapping analyses. The filtered reads were then mapped onto six ASGV genomes, allowing only a single mismatch. Similar analyses were performed for the pooled BRL virus-infected samples. Variant-specific reads were identified as those reads that uniquely mapped (using Bowtie) onto one of the six ASGV genomes, when only allowing perfect matches between the sRNA read and the genome.

### tRF and tRNA-half identification

Mature tRNA sequences of five angiosperms (*Arabidopsis thaliana*, *Brachypodium distachyon*, *Medicago truncatula*, *Oryza sativa* and *Populus trichocarpa*) were retrieved from the PlantRNA database [35]. To identify apple tRFs present, the six NRL datasets were combined and mapped, with Bowtie, onto the retrieved mature tRNA sequences, allowing two mismatches. tRNA-halves were correspondingly identified using the pooled BRL datasets.

### Differential expression analysis of apple sRNA species

The standalone differential expression tool of miRanalyzer [36, 37], which implements the R package, DESeq2 [38], was used to determine variation in sRNA expression levels between the healthy and the ASGV-infected samples. Five distinct sRNA species were investigated using the NRL data, namely miRNAs, phasiRNAs, nat-siRNAs, rasiRNAs and tRFs. The BRL data was used for tRNA-halves differential expression analysis. miRNA analysis was based on miRBase (version 20) [39–42] apple entries, as well as recently predicted novel miRNAs [43]. The phasiRNAs, nat-siRNAs and rasiRNAs analysed were also previously identified [43], while the tRFs and tRNA-haves were identified during the current study. The phasiRNAs included a group of apple tasiRNAs available on the tasiRNAdb [44–46].

### Availability of supporting data

The datasets supporting the results of this article are available in the BioProject repository of the National Centre for Biotechnology Information, BioProject: PRJNA235941 in http://www.ncbi.nlm.nih.gov/bioproject/.

## Electronic supplementary material

Additional file 1: Table S1: tRNA-derived sRNAs identified in the BRL data. The 15 sRNA reads with the highest read counts associated with a tRNA are shown and those differentially regulated (|log2fold change| > =1 and padj < =0.05) as a result of ASGV infection are indicated. **Table S2.** tRNA-derived sRNAs identified in the NRL data. The 15 sRNA reads with the highest read counts associated with a tRNA are shown and those differentially regulated (|log2fold change| > =1 and padj < =0.05) as a result of ASGV infection are indicated. (XLSX 145 KB)

Additional file 2: Table S3: Results for the differential expression analysis of clusters of sRNAs originating from tRNAs, based on BRL data. **Table S4.** Results for the differential expression analysis of the individual tRNA-derived sRNAs, based on BRL data. **Table S5.** Results for the differential expression analysis of clusters of sRNAs originating from tRNAs, based on NRL data. **Table S6.** Results for the differential expression analysis of the individual tRNA-derived sRNAs, based on NRL data. **Table S7.** Results for the differential expression analysis of apple miRNAs present in miRBase. **Table S8.** Results for the differential expression analysis of recently predicted apple miRNAs. **Table S9.** Results for the differential expression analysis of the cluster of nat-siRNAs originating from both strands of the overlapping region of NAT pairs. **Table S10.** Results for the differential expression analysis of the cluster of nat-siRNAs, from the first transcript, originating from the overlapping region of NAT pairs. **Table S11.** Results for the differential expression analysis of the cluster of nat-siRNAs, from the second transcript, originating from the overlapping region of NAT pairs. **Table S12.** Results for the differential expression analysis of all the sRNAs originating from a phased cluster. **Table S13.** Results for the differential expression analysis of phasiRNAs. **Table S14.** Results for the differential expression analysis of tasiRNAs. **Table S15**. Results for the differential expression analysis of the cluster of rasiRNAs originating from both strands of a repetitive sequence. **Table S16.** Results for the differential expression analysis of the cluster of rasiRNAs originating from the forward strand of a repetitive sequence. **Table S17.** Results for the differential expression analysis of the cluster of rasiRNAs originating from the reverse strand of a repetitive sequence. (XLSX 1 MB)

Additional file 3: Table S18: Diagnostic RT-PCR primers. Multiplex-primers used to determine the viral status of apple plants. (XLSX 14 KB)

## Abbreviations

ASGV: *Apple stem grooving virus*
BRL: Broad range library
DCL: Dicer-like
*M*. x *domestica*: *Malus* x *domestica*
miRNA: microRNA
NRL: Narrow rang library
nat-siRNA: Natural-antisense transcript siRNA
NGS: Next-generation sequencing
phasiRNA: phased-siRNA
rasiRNAs: Repeat-associated siRNA
siRNA: Small interfering RNA
sRNA: small RNA
sgRNA: subgenomic RNA
tasiRNA: trans-acting siRNA
tRF: tRNA-derived fragment
vsiRNA: virus-derived siRNA.
